# Emerging Endorobotic and AI Technologies in Colorectal Cancer Screening: A Review of Design, Validation, and Translational Pathways

**DOI:** 10.3390/diagnostics16030421

**Published:** 2026-02-01

**Authors:** Adhari Al Zaabi, Ahmed Al Maashri, Hadj Bourdoucen, Said A. Al-Busafi

**Affiliations:** 1 Human and Clinical Anatomy, College of Medicine and Health Sciences, Sultan Qaboos University, P.O. Box 35, Muscat 123, Oman; 2 Department of Electrical and Computer Engineering, College of Engineering, Sultan Qaboos University, P.O. Box 35, Muscat 123, Oman; 3 Department of Medicine, College of Medicine and Health Sciences, Sultan Qaboos University, P.O. Box 35, Muscat 123, Oman

**Keywords:** colorectal neoplasms, early detection of cancer, robotics, artificial intelligence, computer-aided diagnosis, endoscopy

## Abstract

Advances in artificial intelligence (AI), soft robotics, and miniaturized imaging technologies have accelerated the development of endorobotic platforms that aim to enhance detection accuracy and improve patient experience. In this narrative review, we synthesize evidence on AI-assisted detection and characterization systems (CADe/CADx), robotic locomotion mechanisms, adhesion strategies, imaging modalities, and material and power constraints relating to next-generation CRC screening technologies. Reported performance metrics are interpreted within their original methodological contexts, acknowledging the heterogeneity of datasets, limited representation of diverse populations, underreporting of negative findings, and scarcity of large, real-world comparative trials. We introduce a conceptual translational framework that links engineering design principles with validation needs across in silico, in vitro, preclinical, and clinical stages, and we outline safety considerations, workflow integration challenges, and sterility requirements that influence real-world deployability. Regulatory alignment is discussed using the U.S. FDA Total Product Life Cycle (TPLC) and Good Machine Learning Practice (GMLP) frameworks to highlight expectations for data quality, model robustness, device–software interoperability, and post-market monitoring. Collectively, the evidence demonstrates promising technological innovation but also highlights substantial gaps that must be addressed before AI-enabled endorobotic systems can be safely and effectively integrated into routine CRC screening. Continued interdisciplinary work, supported by rigorous validation and transparent reporting, will be essential to advance these technologies toward meaningful clinical impact.

## 1. Introduction

Colorectal cancer (CRC) is the third most common cancer globally, responsible for over 900,000 deaths annually [1]. In 2020 alone, 1.9 million new cases were diagnosed, reflecting a 30% increase in disease burden over the past decade [2]. Projections suggest a further 3% annual rise in incidence over the next two decades, highlighting the urgent need for effective and accessible screening strategies [2]. Early detection through colonoscopy and other screening programs can reduce CRC mortality by up to 70% while also lowering healthcare costs associated with late-stage diagnosis and treatment [3,4]. Despite its benefits, widespread adoption of current screening methods remains limited due to multiple systemic and procedural barriers.

Several CRC screening modalities exist, ranging from non-invasive to invasive techniques [5]. Current methods include fecal occult blood tests (FOBT), fecal immunochemical tests (FIT), flexible sigmoidoscopy, and colonoscopy. These methods focus on detecting colonic polyps, benign lesions with potential for malignant transformation if not removed [6,7]. Among these, colonoscopy is considered the gold standard due to its dual diagnostic and therapeutic capabilities. It enables complete visualization of the colon, removal of precancerous lesions, and tissue biopsy [5,8,9,10]. However, its uptake is limited by patient discomfort, the need for sedation, socioeconomic disparities, and challenges with bowel preparation—all of which reduce compliance and lead to incomplete examinations [11,12,13]. These issues are compounded by the high costs of specialized equipment and limited availability of trained personnel, particularly in underserved regions [14,15,16]. These constraints have motivated the development of artificial intelligence (AI) tools and endorobotic platforms intended to improve lesion detection, reduce procedural discomfort, and support more standardized diagnostic quality.

Significant progress has been made in AI-assisted polyp detection (CADe), histologic characterization (CADx), and advanced imaging modalities, as well as in soft robotic locomotion, adhesion mechanisms, and miniaturized embedded systems. However, despite encouraging results from controlled experimental and early clinical studies, the evidence base remains heterogeneous, with limited representation of diverse patient populations, few large prospective trials, and a notable scarcity of real-world performance data [17]. Published results often report high sensitivity or negative predictive values, but these metrics derive from distinct datasets, imaging conditions, and validation protocols; without consistent methodological context, performance comparisons may be misleading. Furthermore, many studies do not report neutral or negative findings, contributing to publication bias and making it difficult to assess true clinical benefit [18,19,20]. Recent gastroenterology task forces have emphasized the need for standardized metrics, multicenter validation, and governance frameworks to support safe and effective integration of AI into endoscopic workflows [18].

To clarify the evidentiary scope of this manuscript, we emphasize that this work is intentionally structured as a narrative, technology-focused review. The available evidence is highly heterogeneous, ranging from benchtop prototypes and preclinical systems to early feasibility trials and exploratory AI models. As a result, formal systematic-review methods such as PRISMA workflows, risk-of-bias scoring, or quantitative meta-analysis are not well suited to this evidence base due to its heterogeneity and early developmental focus. The objective of this review is to synthesize the technological, clinical, and translational dimensions of emerging AI-enabled endorobotic innovations. To achieve this, we conducted a non-systematic targeted narrative search using PubMed, IEEE Xplore, and Scopus (2009–2024) to identify representative contributions that advance understanding of system design, AI performance requirements, or translational considerations, including human feasibility trials, animal and ex vivo experiments, and simulation or phantom-based studies. Studies were selected based on relevance to screening, technological innovation, and contribution to translational or validation insight rather than exhaustive coverage or predefined Boolean syntax. While early foundational studies are cited to contextualize the evolution of endorobotic and AI-assisted endoscopy, the critical synthesis and translational analysis in this review emphasize developments from 2020 to 2025, reflecting the period of rapid clinical translation and regulatory engagement. Representative literature was identified using flexible combinations of terms related to colonoscopy robotics, capsule endoscopy locomotion, soft robotic endoscopy, AI-assisted polyp detection (CADe), and optical biopsy (CADx), consistent with a narrative review approach. Mature cost-effectiveness analyses, regulatory-grade safety datasets, and population-level generalizability studies remain limited; therefore, these components are addressed conceptually rather than through comparative statistics.

Recent reviews have extensively examined AI-assisted colonoscopy and colorectal cancer management, yet important translational gaps remain. Most colonoscopy-focused reviews emphasize CADe and CADx performance, randomized trial evidence, and clinical outcomes, while largely overlooking the engineering foundations of endorobotic systems such as locomotion, adhesion, power delivery, and material constraints and how these factors influence preclinical validation and clinical readiness [21,22,23]. Broader CRC and screening reviews synthesize AI applications across diagnosis and treatment, including robotic surgery, but typically treat endoscopic AI, robotic platforms, and imaging technologies as separate domains with limited linkage to device-level regulatory pathways [24,25,26,27].

The present review addresses these gaps by proposing a clinically grounded translational framework for AI-enabled endorobotic colorectal cancer screening that integrates subsystem interdependencies (locomotion–adhesion–imaging–power–materials), organizes heterogeneous evidence across in silico, bench, preclinical, and early human feasibility stages, and aligns technological maturation with contemporary total product lifecycle and good machine learning practice expectations.

This review examines the emerging landscape of endorobotic and AI-assisted technologies for CRC screening, key engineering principles, validation strategies, and regulatory considerations. Specifically, this review addresses the following questions: (i) what engineering and AI design principles currently underpin endorobotic CRC screening systems; (ii) how these systems have been validated across experimental, preclinical, and early clinical settings; and (iii) which translational and regulatory barriers most limit real-world adoption. We propose a conceptual translational framework to help contextualize how engineering advances, clinical requirements, and regulatory expectations interact along the path toward clinical deployment. Particular attention is given to maturity of systems, manufacturability, mucosal safety, sterility and workflow integration, economic feasibility, and alignment with FDA Total Product Life Cycle (TPLC) and Good Machine Learning Practice (GMLP) principles.

Although interdependencies among locomotion, adhesion, imaging, power delivery, computation, and AI inference are highlighted, current evidence does not support a full data-driven case study of a complete endorobotic system. Safety metrics and multicenter AI validation remain sparse. Overall, this narrative review aims to provide clinicians, researchers, and developers with a balanced and realistic understanding of the opportunities and limitations shaping next-generation CRC screening technologies, with emphasis on what current systems can achieve under controlled conditions and what remains necessary to enable safe, scalable, and clinically meaningful adoption.

**Figure 1:**
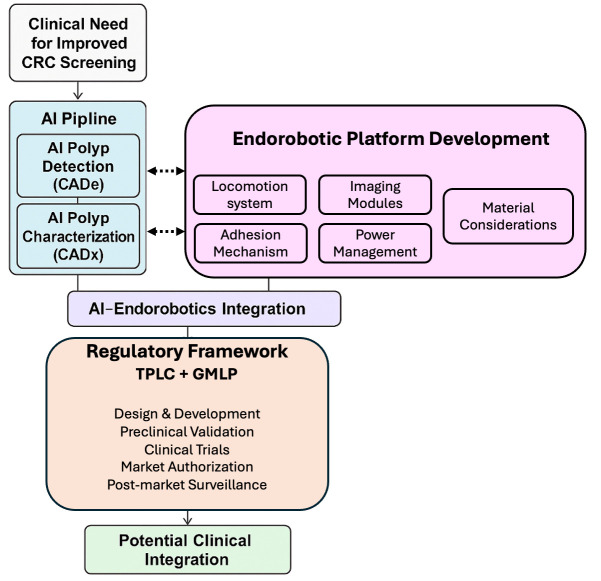
Conceptual framework illustrating the key components involved in developing AI-enabled endorobotic systems for colorectal cancer screening, including the AI pipeline, endorobotic platform design, regulatory considerations, and toward clinical integration.

Figure 1 summarizes the conceptual framework guiding this review, outlining how clinical need drives the parallel development of AI pipelines and endorobotic subsystems before progressing through integration, validation, regulatory alignment, and eventual clinical translation.

## 2. Technological Innovations in Colorectal Cancer Screening

### 2.1. Artificial Intelligence in Colorectal Cancer Screening

Artificial intelligence (AI) has become a central component of innovation in colorectal cancer (CRC) screening, particularly through its integration into colonoscopy and CT colonography workflows. Most existing systems focus on computer-aided detection (CADe) and computer-aided diagnosis (CADx), with the goal of supporting endoscopists by improving lesion recognition, reducing perceptual errors, and enabling greater diagnostic consistency. However, despite promising performance in controlled studies, real-world validation remains limited and reported accuracy metrics must be interpreted within their specific methodological contexts.

#### 2.1.1. AI for Polyp Detection (CADe)

Adenoma detection rate (ADR) is a key performance metric in CRC screening, defined as the ratio of patients who have at least one colorectal adenoma identified during their initial screening colonoscopy to the total number of patients evaluated by an endoscopist. Each 1% increase in ADR linked to a 3% reduction in interval CRC risk [28]. Early landmark systems employed convolutional neural networks (CNNs) for real-time detection, achieving high sensitivity and specificity during colonoscopy [29,30,31].

Several commercially available systems have demonstrated high sensitivity for pre-neoplastic lesions. For instance, GI Genius, a real-time CNN-based platform, achieved 99.7% sensitivity in detecting polyps during colonoscopy [32]. Similarly, EndoBRAIN, outperformed experienced endoscopists achieving a sensitivity of 96.9%, specificity of 100%, and an overall accuracy of 98%. It was particularly effective at distinguishing neoplastic from non-neoplastic lesions, with corresponding sensitivity and specificity values of 96.9% and 94.3% respectively [33]. It is worth mentioning that these systems primarily improve detection of diminutive polyps (<5 mm), which are often hyperplastic and carry low malignant potential. Detection of larger, clinically significant adenomas remains only marginally improved. Moreover, increased false positives may lead to unnecessary resections, raising concerns about cost-effectiveness and patient safety [29,34] (Table 1).

Recent studies have begun to explore more advanced architectures, including hybrid attention-based models and early transformer-based implementations, although these remain largely investigational and lack multi-center validation.

CADe performance is strongly influenced by the quality and diversity of the datasets used for model development. Most published systems are trained on single-center colonoscopy datasets, which limits demographic diversity and increases the risk of overfitting to specific imaging conditions or endoscope types. Several evaluations have highlighted substantial heterogeneity in annotation practices, lesion prevalence, and dataset composition, with many datasets enriched for diminutive polyps or curated high-quality frames rather than full-motion colonoscopy videos [35]. More recently, multi-institutional benchmarking using open-access datasets has reinforced the challenge of dataset generalizability across centers [36]. These limitations collectively challenge the generalizability of CADe models, particularly when deployed across different colonoscopy platforms or patient populations.

In addition to dataset constraints, real-time feasibility remains a key barrier for widespread clinical integration. Although CADe systems have demonstrated promising per-frame detection performance under controlled conditions, real-world deployment depends on computational efficiency, hardware acceleration, and stable processing throughput during high-resolution video acquisition. A recent benchmarking study comparing multiple commercially available CADe software reported statistically significant differences in sensitivity, false-positive rates, and alert delay among systems, underscoring the need for standardized performance metrics and embedded-system optimization [20]. Moreover, many published CADe models do not report inference times or embedded-hardware performance, and most rely on workstation-level GPUs, highlighting the ongoing challenge of integrating advanced AI algorithms into miniaturized endoscopic processors and video towers [37].

Public colonoscopy datasets such as Kvasir SEG, CVC ClinicDB, and HyperKvasir are widely used to train and benchmark polyp detection and segmentation models and have enabled extensive cross-dataset generalization studies in related work. However, these collections are typically derived from a limited number of centers, involve relatively small patient cohorts, and often rely on curated still frames rather than complete procedures, which can introduce selection bias and constrain real-world generalizability [38,39].

#### 2.1.2. AI for Polyp Characterization (CADx)

Not all polyps detected during a colonoscopy are preneoplastic or require removal. Accurate characterization is essential to distinguishing high-risk lesions from benign ones as misclassification can lead to unnecessary interventions, increasing both healthcare costs and patient burden. Endoscopists accuracy for identifying and categorizing small polyps (less than 5 mm) remains below 80%, highlighting a critical gap in diagnostic performance [40].

While CADe systems enhance detection, Computer-Aided Diagnosis (CADx) systems provide a real-time histologic classification of detected polyps. These tools aim to support “diagnose-and-leave” or “resect-and-discard” strategies, potentially reducing unnecessary polypectomies and histopathological assessments.

A prominent example is the ENDOANGEL system, a deep neural network trained to analyze mucosal and vascular features of lesions. In a randomized controlled trial, ENDOANGEL improved adenoma detection rate from 8% (standard colonoscopy) to 16%, demonstrating significant value as a real-time decision-support tool [41]. A related system, ENDOANGEL-CPS, estimated polyp size with a relative accuracy of 89.9%, markedly exceeding the 54.7% accuracy achieved by endoscopists [42]. Complementing these developments, other convolutional neural network (CNN) models have achieved a 97% negative predictive value for distinguishing adenomas in diminutive rectosigmoid polyps [43]. This advancement directly supports more precise surveillance recommendations and risk stratification (Table 1).

Most clinically evaluated CADx systems for optical biopsy of diminutive colorectal polyps still rely on convolutional neural networks (CNNs) trained on narrow-band imaging or endocytoscopy data [44,45]. More recently, several investigational systems have begun to incorporate attention mechanisms and transformer-style encoders for colonoscopic image analysis, particularly for polyp segmentation and early CRC detection. These architectures includes ViT-based polyp segmentation frameworks such as UViT-Seg [46], PolySegNet, which fuses Swin and vision transformers [47], and ViTCol/PUTS for transformer-based classification of endoscopic pathological findings and polyp segmentation [48]. These architectures exploit self-attention to capture long-range contextual information but generally require larger annotated datasets and higher computational budgets than conventional CNNs, as highlighted in recent analyses of transformer-based polyp segmentation models, which note their increased data and memory demands compared with U-Net-like CNNs [49]. These factors may limit their suitability for embedded or edge deployment in endoscopic systems without model compression or hardware acceleration.

#### 2.1.3. AI in CT Colonography (Virtual Colonoscopy)

Virtual colonoscopy, or CT colonography (CTC), is a non-invasive imaging alternative used primarily for patients unwilling or unable to undergo traditional colonoscopy. AI models applied to CTC assist in detecting subtle lesions such as flat polyps that are frequently missed by conventional endoscopy.

In a recent study, Grosu et al. developed a machine learning algorithm that achieved 82% sensitivity and 85% specificity in differentiating benign from precancerous lesions [50]. Similarly, Taylor et al. demonstrated that polyp detection sensitivity decreased with reduced sphericity, highlighting a key limitation of current models [40].

Although promising, AI-driven CTC remains supplementary, not substitutive. High cost, radiation exposure, and the need for follow-up colonoscopy in positive cases limit its widespread utility. Despite clinical adoption of CT colonography, AI-driven CTC tools workstation-based, rely on proprietary datasets, and lack standardized real-time performance benchmarks, limiting their scalability across routine screening workflows. Even where CAD tools exist, they have not displaced colonoscopy as the primary screening modality and lack widespread regulatory endorsement for standalone screening Table 1.

**Table 1:** Comparative overview of Endotics, colon capsule endoscopy, and conventional colonoscopy for colorectal evaluation.

Feature	CADe (Detection) [32,42,51]	CADx (Characterization) [44,51]	AI in CT Colonography (CTC) [50,52,53]
Primary Function	Real-time detection of polyps during colonoscopy.	Real-time histologic/optical classification of polyps (“virtual histology”)	Non-invasive identification of Colorectal lesions from CT imaging.
Key Metrics	Sensitivity up to ~95–100% in image/ polyp-level validation and real-time testing (e.g., GI Genius–type systems)	Negative predictive value (NPV) up to 97% for diminutive polyps (e.g., ENDOANGEL-CPS).	CTC polyp characterization reach AUC 0.83–0.91 with sensitivities ~80–82% and specificities ~69–85%
Clinical Benefit	Increases adenoma detection rate (ADR); reduces missed lesions.	Supports “resect and discard” and “diagnose and leave” strategies, potentially lowering pathology workload.	Provides an option for patients unwilling or unable to undergo colonoscopy.
Limitations	High false-positive rate; strongest effect on diminutive/non-advanced adenomas	Model bias; limited accuracy for sessile/flat lesions and external generalizability concerns	Involves ionizing radiation; CT colonography positive (and AI-flagged) findings still require colonoscopy for biopsy/resection
Regulatory Status	FDA-approved (e.g., GI Genius).	Mostly investigational or early clinical; few systems approaching regulatory pathways	In proof-of-concept and validation phases; no FDA-approved
Readiness Level	Available for clinical use in select settings.	Early-stage clinical trials and pilot implementations; limited routine uptake	Experimental/adjunct only; requires further validation.

#### 2.1.4. Limitations of Current AI Models

**Figure 2:**
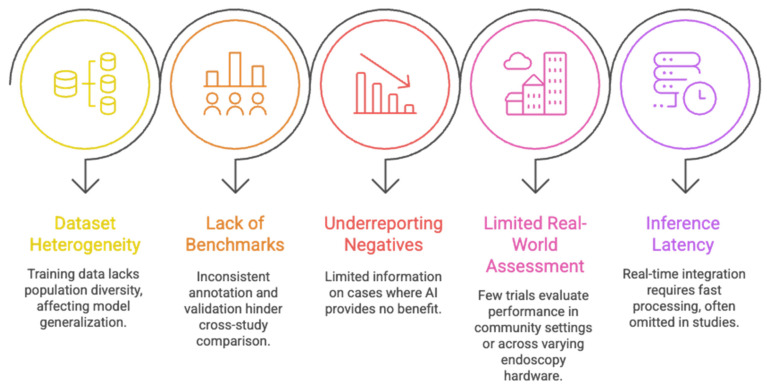
Key limitations hindering the clinical translation of AI systems in colorectal cancer screening.

Current AI systems for colorectal cancer screening face several important limitations that restrict their generalizability and readiness for real-world deployment Figure 2. Training datasets are often heterogeneous and lack adequate population diversity, limiting model robustness across different clinical settings. Benchmarking remains inconsistent, with variable annotation standards and validation protocols that hinder meaningful comparison across studies. Negative or neutral results are frequently underreported, contributing to publication bias and an incomplete understanding of model performance. Moreover, few systems have been evaluated in community-based or resource-variable environments, creating a gap between controlled study performance and everyday clinical reality. Finally, reliable real-time deployment depends on low-latency inference, yet many studies do not report computational constraints or the impact of hardware variability on frame-rate performance including available power budgets, thermal dissipation, memory footprint, and sustained frame-rate requirements. Furthermore, Human-in-the-loop considerations, including operator trust and reduced tactile feedback in robotic systems, remain underexplored and warrant dedicated human-factors research. Collectively, these gaps highlight the need for more rigorous, transparent, and representative evaluation before AI tools can be confidently integrated into routine colorectal cancer screening practice [19].

#### 2.1.5. Regulatory Landscape for AI in CRC Screening

The regulatory approval of AI-based tools for gastrointestinal (GI) endoscopy remains selective and challenging. As of mid-2024, only 13 out of 882 FDA-cleared AI/ML-based medical devices are dedicated to GI endoscopy. This reflects the stringent safety, efficacy, and generalizability criteria required for clinical use.

Most cleared systems are CADe models, reflecting the lower regulatory risk of detection compared to diagnosis. CADx tools, which influence real-time clinical decision-making, face higher evidentiary requirements, including demonstration of safety, consistency, and generalizability. A notable success is GI Genius (Medtronic), the first FDA-approved AI platform for real-time polyp detection [54]. Its clearance in 2021 was supported by randomized clinical data demonstrating increased adenoma detection rates. This sets a precedent for CADe models, especially those focused on detection rather than diagnosis.

Similarly, AI tools for CT colonography are currently limited to research environments. These require not only image analysis validation but also assurance that findings lead to actionable interventions (i.e., follow-up colonoscopy).

As the FDA transitions toward adaptive regulatory frameworks for AI, future approvals may rely more on post-market surveillance and real-world performance data. To support this, robust multi-center trials and harmonized outcome metrics are essential [18].

### 2.2. Robotic-Assisted Colonoscopy Platforms

**Figure 3:**
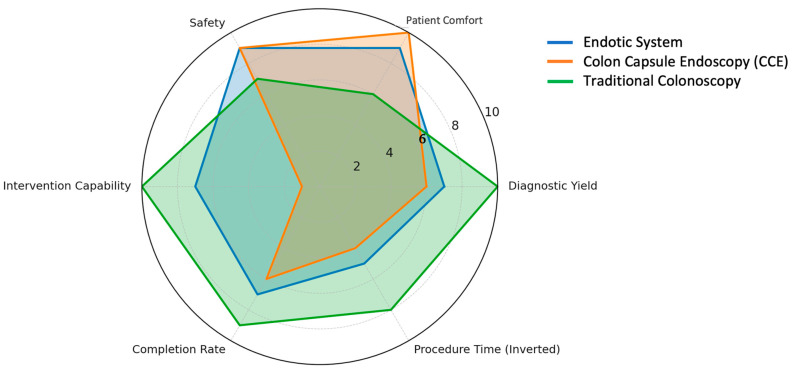
Comparative radar chart of colonoscopy technologies.

Robotic-assisted colonoscopy (RC) platforms seek to overcome the limitations of conventional colonoscopy (CC), such as patient discomfort, incomplete exams, and dependence on skilled operators. Existing systems vary widely in locomotion strategy, adhesion mechanisms, actuation technologies, and degree of autonomy. However, evidence remains limited, and many performance metrics derive from prototype-level or early feasibility studies rather than large comparative trials. As such, reported results should be interpreted within their original methodological and clinical contexts. Accordingly, comparative tables throughout this review emphasize qualitative differentiation of system design, locomotion strategy, study context (ex vivo, in vivo, early feasibility), and translational maturity, rather than quantitative performance metrics, which are inconsistently reported or not comparable across prototype-level studies. Table 2 and Figure 3. For clinical translation, robotic endoscopic platforms must demonstrate not only locomotion feasibility but also mucosal safety, sterility compatibility, procedural reliability, and alignment with existing endoscopy workflows.

#### 2.2.1. Clinical-Ready and Near-Clinical Platforms

##### Endotics System (E-Worm)

The Endotics system is among the few soft robotic colonoscopy platforms evaluated in clinical settings. Using a biomimetic peristaltic motion, it advances through sequential anchoring and elongation cycles designed to reduce pain and avoid looping. Clinical studies report high patient comfort and low mucosal injury rates, although interpretation is constrained by small sample sizes and operator learning effects. Cecal intubation rates of approximately 80% have been reported, but these remain lower than rates achieved with conventional colonoscopy performed by experienced endoscopists. Procedure times are typically longer, and the platform is highly sensitive to bowel preparation quality [55].

##### Colon Capsule Endoscopy (CCE)

CCE offers a completely non-invasive alternative in the form of a swallowable imaging capsule. Although patient acceptance is high and mucosal trauma is virtually eliminated, CCE lacks therapeutic capability and requires excellent bowel preparation. Sensitivity for polyps ≥6 mm is moderate, and any positive finding necessitates referral to conventional colonoscopy, limiting workflow efficiency. Furthermore, image analysis takes 8–16 h, and any positive finding mandates a follow-up colonoscopy [56]. As with other technologies, performance varies across studies, and negative or neutral results—such as missed sessile serrated lesions—are not consistently reported.

**Table 2:** Comparative Overview of AI Applications in Colorectal Polyp Assessment and CT Colonography.

Feature	Endotics System (E-Worm) [57,58,59]	Colon Capsule Endoscopy (CCE) [56,60]	Traditional Colonoscopy [16,61]
Invasiveness	Minimally invasive; self-propelled soft probe.	Non-invasive; swallowed capsule.	Invasive; requires endoscope insertion.
Interventional Capability	Yes—allows biopsy and polyp removal.	No—diagnostic only.	Yes—biopsy, polypectomy, and therapeutic procedures.
Patient Comfort	High; no sedation required.	Very high; no sedation or manipulation.	Moderate; sedation typically required.
Completion Rate	~82% cecal intubation.	Variable (~70% in some studies).	>90% with experienced endoscopists.
Diagnostic Accuracy	~93% for polyps ≥6 mm.	~87% sensitivity, 88% specificity for ≥6 mm polyps.	>95% for polyps >10 mm.
Reusability	Single-use disposable.	Single-use capsule.	Reusable (requires reprocessing).
Procedure Duration	~45 min.	10–14 h (transit), plus 30–60 min review.	30–60 min.
Safety Profile	Excellent; minimal mucosal trauma, no sedation risk.	Very safe; rare capsule retention.	Generally safe, but sedation-related risks.
Power Source	Tethered.	Internal battery.	Tethered/manual.
Current Limitations	Sensitive to bowel prep; slower procedure.	No therapeutic capability; prolonged excretion time.	Discomfort, sedation risks, high operator skill requirement.

#### 2.2.2. CAD Clinical Readiness

To examine how far current technologies have progressed toward clinical use, we evaluated the maturity of existing AI-enabled and endorobotic systems within the context of colorectal cancer screening. Although Technology Readiness Levels (TRLs) are often applied to characterize the development of biomedical devices, they are not directly applicable in this domain because most endorobotic and AI platforms lack the standardized validation, multicenter testing, and regulatory benchmarking required for formal TRL scoring. Instead, we adopt a qualitative classification that more accurately reflects the heterogeneity of the available evidence, describing maturity across three developmental stages: prototype development, preclinical evaluation, and early human feasibility.

Using this framework, real-time CADe systems represent the most clinically advanced group, supported by multicenter randomized trials, regulatory clearances, and demonstrated improvements in adenoma detection in routine practice. CADx systems occupy an intermediate position, with strong performance in controlled settings but limited generalizability and ongoing regulatory review. In contrast, endorobotic platforms such as pneumatic or inchworm locomotion systems, magnetically steered capsules, and soft robotic devices remain largely in preclinical or early feasibility phases. Key translational barriers include incomplete safety profiling, sterility and reprocessing workflows, manufacturability constraints, and the absence of large, prospective or real-world datasets. Additionally, few systems meet the documentation standards needed for regulatory submissions, such as consistent reporting of performance metrics, human-factors validation, cybersecurity safeguards, and post-deployment monitoring plans. Until these evidence, safety, and regulatory milestones are met, the transition from promising prototypes to scalable clinical tools will remain limited [22,62].

#### 2.2.3. Challenges in Robotic-Assisted Colonoscopy

Despite promising advancements, robotic-assisted colonoscopy (RC) systems face several challenges that limit their scalability, cost-effectiveness, and clinical integration. These challenges are multidimensional, encompassing technical, operational, economic, and regulatory barriers as the following:

A. Technical Challenges

1. Navigation and Locomotion Efficiency

The human colon presents a tortuous, deformable, and friction-variable environment. Robotic systems must traverse long distances with sharp bends and inconsistent lumen diameters. Unlike conventional colonoscopes, which are operator-controlled in real time, robotic systems must rely on embedded navigation strategies or external manipulation (e.g., magnetic control). Maintaining reliable forward progression without inducing mucosal injury or looping remains a primary challenge.

2. Adhesion Mechanisms

Maintaining stable adhesion to the mucosal surface is essential for maneuverability and visual stability. However, existing mechanisms such as suction, micro-hooks, or magnetic control each have trade-offs related to tissue trauma, detachment risk, or external hardware complexity.

3. Imaging and Visualization

High-resolution imaging is critical for polyp detection, yet robotic systems must house miniaturized sensors that are energy-efficient and robust in low-light, fluid-filled conditions. Adding advanced imaging (e.g., NBI, multispectral) further increases design and energy demands.

B. Operational Constraints

1. Bowel Preparation Sensitivity

Robotic systems are often more sensitive to residual stool or fluid than traditional colonoscopes. Obstructed viewports and locomotion clogs can lead to procedure termination.

2. Tether Management and Mobility

While wireless systems are ideal, most current designs rely on soft tethers for power and data transmission, which introduce drag, reduce maneuverability, and increase mucosal friction.

C. Economic and Regulatory Barriers

1. High Manufacturing Costs

Although economic and workflow considerations are critical for adoption, the current literature provides no quantitative or standardized cost-effectiveness analyses for endorobotic colonoscopy systems. Available studies remain limited to early feasibility trials without reporting capital costs, disposable requirements, procedure time, throughput, staffing implications, or validated economic endpoints. As such, any economic discussion remains conceptual, and robust cost-effectiveness modeling must await multicenter clinical trials and real-world deployment. In general, advanced robotic systems require custom fabrication, biocompatible materials, and high-precision assembly, resulting in elevated per-unit costs compared to reusable conventional colonoscopes.

2. Lack of Standardized Evaluation Frameworks

The absence of universally accepted performance benchmarks or regulatory pathways delays approval and reimbursement. Comparative clinical trials are limited.

To address these technical and clinical challenges, the following section outlines the engineering design framework underpinning endorobotic colonoscopy platforms.

## 3. Engineering Design Framework for Endorobotic Colonoscopy Systems

The development of endorobotic systems for colorectal cancer screening requires an integrated engineering approach that aligns locomotion, adhesion, imaging, power, and material considerations while ensuring patient safety and regulatory feasibility. Although numerous innovative prototypes have been proposed, few have reached advanced clinical testing, in part because subsystem trade-offs are often optimized in isolation rather than through a system-level design process. This section outlines the major engineering dimensions and highlights the interdependencies, risks, and translational constraints that influence real-world feasibility.

### 3.1. Endorobotic Locomotion Systems and Safety Challenges

Locomotion is fundamental to device navigation in the colon, a highly deformable and tortuous environment. Existing platforms employ peristaltic, ambulatory, wheeled/rolling, magnetic, or passive modes of movement, each associated with specific advantages and risks. Table 3 compares these modalities.

I. Peristalsis locomotion

Inspired by earthworm-like motion, this method uses segmental contraction for smooth propulsion. It is particularly suited to the narrow, tortuous colon due to its tissue-conforming design. The segmented body allows precise motion control, reducing trauma risk.

Despite these advantages, the design and implementation of peristaltic locomotion present several engineering challenges. One of the primary concerns is achieving the optimal balance between flexibility and stiffness in each segment. Excessive rigidity may lead to tissue damage, while excessive softness can compromise locomotion efficiency. Moreover, the robot must maintain consistent and adaptive movement against variable resistive forces exerted by the colon walls. This necessitates sophisticated mechanical design and actuation systems capable of dynamic response [61,63].

Recent soft-robotic designs employ soft silicone-based actuators for safe interaction with the colonic mucosa and a tendon-driven control system to enable low-force, yet precise, propulsion. Demonstrated in both ex vivo and in vivo models, this design showed effective navigation while significantly reducing patient discomfort [64].

II. Ambulatory (Legged) Locomotion

This approach is inspired by biological organisms that walk or crawl using limbs. A primary benefit of ambulatory robots lies in their precision and adaptability—key qualities that allow for fine-tuned navigation through convoluted intestinal pathways [65]. Their limb-based architecture enables them to adjust dynamically to irregular surfaces and obstacles, reducing the likelihood of unintended tissue trauma during procedures [66].

Despite these advantages, the development of ambulatory endorobots presents considerable challenges. The mechanical complexity required to produce multiple articulating limbs capable of synchronized motion introduces significant engineering hurdles. These include difficulties in miniaturization, actuation, and control, which collectively raise manufacturing costs and complicate maintenance [64,67]. Moreover, the direct physical interaction of the robot’s limbs with the colonic mucosa increases the potential for mechanical irritation or injury, posing a safety concern that must be carefully addressed in design [66].

Although currently less common than other modes of locomotion, ambulatory endorobots remain an active area of research. Innovations continue to focus on refining limb design, enhancing locomotion efficiency, and integrating advanced control systems to improve safety [66].

III. Wheeled/Rolling Locomotion

Rolling or wheeling-based locomotion in endorobotics is inspired by vehicular motion, employing wheels, tracks, or similar mechanisms to traverse the gastrointestinal tract. This mode of propulsion offers distinct advantages, particularly in terms of locomotion efficiency, speed, and platform stability, making it well-suited for diagnostic and therapeutic procedures requiring precision and time efficiency. By maintaining a continuous contact surface and leveraging rolling dynamics, these systems can potentially move through the colon more rapidly than peristaltic or ambulatory robots. However, its effectiveness is reduced in angulated or collapsed colonic segments, where rigid components may generate excess pressure, increasing injury risk. Current research focuses on enhancing wheel flexibility and traction materials to address this [63].

IV. Immobile (Passive) platform

In the context of endorobots, the term immobile refers to devices that do not generate their own locomotion within the gastrointestinal tract. Instead, these systems rely on external forces such as manual manipulation by a colonoscopist or externally applied magnetic fields for movement and positioning. They reduce onboard complexity and may enhance safety, but their motion is limited by magnetic field resolution and anatomical variability [68]. Devices like the Magnetic Tentacle Robot and STIFF-FLOP have shown potential but struggle with precise control in narrow, spasmodic, or collapsed segments [69]. Other systems, such as those employing autonomous lumen-tracking algorithms, have shown vulnerability to delays in haptic feedback—often exceeding 200 milliseconds—which can cause overshooting or unintentional collisions. In complex anatomical settings, such delays necessitate manual override, compromising the autonomous capabilities of the robot [70]. These systems are often used in simpler diagnostic procedures, like capsule endoscopy, where the device passively travels through the digestive tract. The current development focuses more on improving external control methods to enhance navigation accuracy and safety.

V. AI/ML-Assisted Navigation and Control in GI Endorobotics

It is worth mentioning that reinforcement learning (RL) and other advanced control algorithms are increasingly explored for autonomous steering, path planning, and stabilization of endorobotic devices within the colon. Early work by Trovato et al. demonstrated that Q-learning and SARSA-based controllers could adaptively regulate propulsion and contact forces in a robotic colonoscope navigating deformable and slippery colonic environments [71]. More recent studies have applied deep visuomotor reinforcement learning for endoscopic navigation, enabling AI systems to interpret visual cues and predict optimal steering actions in simulated colonoscopy tasks [72]. Safety-constrained RL frameworks have also been proposed, combining policy optimization with formal verification methods to prevent unsafe maneuvers during colon navigation [73].

However, these approaches remain confined to simulation environments, benchtop phantoms, or small-scale user studies. They rely on simplified models of colonic biomechanics and have not yet been evaluated in human subjects. Consequently, RL-driven autonomy should be regarded as an early-stage research direction promising but not mature and is likely to complement rather than replace human-in-the-loop control in future endorobotic platforms.

### 3.2. Adhesion Mechanisms and Mucosal Safety Considerations

Adhesion is vital for traction, stabilization, and force transmission in endorobotics systems. In addition to supporting locomotion, robust and controllable adhesion is fundamental for therapeutic intervention. Stable wall anchoring enables precise orientation for tasks such as targeted biopsy, controlled polyp resection, mucosal lifting, and localized drug delivery. Insufficient adhesion or slippage can compromise procedural accuracy and increase the risk of mucosal injury, underscoring its central role in both diagnostic and therapeutic endorobotic applications.

The key challenge is achieving sufficient grip while minimizing mucosal trauma. Four principal adhesion strategies are used, each with translational trade-offs as presented in Table 4:

I. Suction adhesion

Suction-based mechanisms utilize tubes or cups to create negative pressure against the mucosal wall, enabling firm yet adaptive grip for navigation. Suction offers improved traction with potentially reduced mechanical trauma compared to rigid contact methods [74]. However, the colon’s dynamic environment—characterized by peristalsis and mucosal folds (haustra)—complicates the adhesion dynamics. Excessive suction may damage delicate tissues, while insufficient suction compromises maneuverability. Ongoing research focuses on optimizing suction force and biomaterial compatibility to enhance safety and efficiency during colonoscopy procedures.

II. Microhooks and Barbs

Mechanical hooks or barbs enable secure anchorage to the colon wall. This approach provides robust grip, enhancing stability and locomotion, but poses a higher risk of mucosal injury. Hence, precision in deployment and control is critical. Current advancements aim to develop miniaturized, soft, and biocompatible hook designs that maintain traction while reducing trauma [74].

III. Adhesive pads

These systems employ bioinspired sticky surfaces to achieve gentle, reversible adhesion. Adhesive pads offer smoother navigation and reduced injury risk; however, concerns persist regarding residual materials that may interfere with diagnostics. Research is directed at developing smart adhesives with tunable adhesion strength and clean detachment properties for safe mucosal interaction.

IV. Magnetic systems

Magnetic systems utilize external magnetic fields to guide and adhere endorobots non-invasively. This method minimizes direct tissue contact, reducing trauma while enabling precise remote navigation [66]. Limitations include the need for external magnetic setups and restricted operational range. Innovations focus on improving magnetic field modulation and miniaturization to enhance clinical applicability.

The lack of quantitative measurements of adhesion force, slip thresholds, and mucosal tolerance across devices remains a barrier to standardization.

Although individual experimental studies report adhesion forces or pressure thresholds under controlled conditions, the absence of standardized testing protocols and comparable tissue models precludes meaningful cross-platform quantitative comparison at this stage.

**Table 4:** Comparative Characteristics of Adhesion Mechanisms in Endorobotics.

Adhesion Mechanism	Grip Strength	Tissue Safety	Reversibility	External Dependency	Translational Implications
Suction	Moderate	Moderate (risk of mucosal trauma with prolonged use)	High	No	Simple and effective; widely used but limited by potential tissue injury and air leakage in dynamic environments.
Microhooks	High	Low (risk of mucosal penetration)	Moderate	No	Provides strong anchoring but poor biocompatibility; limited clinical feasibility.
Adhesive Pads	Low–Moderate	High	High	No	Biocompatible and reversible; promising for short-term adhesion but limited durability in wet/mucosal environments.
Magnetic Coupling	Moderate	Very High	High	Yes (requires external magnetic field/guidance system)	Enables atraumatic adhesion with excellent safety; external hardware is a barrier to portability and scalability.

### 3.3. Imaging and Sensing Constraints in Endorobotic Platforms

High-resolution imaging technologies have become essential in enhancing the detection of early colorectal abnormalities, particularly flat or small lesions such as sessile serrated and diminutive adenomas under 5 mm in size. Advanced image sensors in high-definition (HD) colonoscopy improve the clarity of mucosal surfaces and vascular patterns, allowing for more accurate visualization Table 5. This improvement has been associated with a 15–25% increase in adenoma detection rates compared to standard-definition systems. Moreover, the integration of artificial intelligence (AI) into these imaging platforms further boosts diagnostic precision, achieving sensitivity rates exceeding 95% for lesions smaller than 3 mm. These advancements collectively support earlier and more reliable identification of precancerous conditions during routine screenings [75,76]. Despite this advancement, no platform to date has demonstrated consistent performance in variable real-world clinical conditions.

### 3.4. Power Management and System Stability

Power management remains a critical design consideration in mobile colon endorobots, particularly for wireless systems constrained by internal batteries and thermal thresholds. While tethered robotic systems typically draw 5–15 W of power, wireless capsule endorobots must operate within a 0.5–3 W budget to sustain diagnostic and locomotion functions over procedures lasting 20 to 60 min. Component-level consumption data show that micromotor-based propulsion can require up to 2 W, HD CMOS sensors consume approximately 0.5 W, and ARM-based microcontrollers operate near 0.3 W. Optional AI integration may raise demands significantly (5–10 W), while wireless transmission and real-time video streaming each contribute 0.5–2 W depending on implementation as shown in Table 6. These figures highlight the need for careful integration of low-power electronics in future endorobotic platforms. Locomotion strategies influence power budgets—rolling systems require higher torque, while peristaltic robots can use tendon-driven low-energy actuators. Similarly, strong suction-based adhesion may demand additional actuation power, impacting battery requirements and thermal loads.

Video streaming and AI-based diagnostics significantly increase energy needs. Advanced energy-efficient components and low-power protocols are under investigation to overcome these limitations.

Battery selection in endorobotic systems is driven by the need for high energy density, safety, and compact integration. Lithium carbon monofluoride (Li/CFx) cells are widely used for their high energy capacity (up to 720 Wh/kg), long shelf life, and chemical stability. These features make them well-suited for wireless capsule endoscopy and other mobile diagnostics, although their discharge kinetics and heat generation limit performance at high rates. Lithium iodide batteries are another common solution, especially in implanted medical devices, due to their long-term reliability and leak-proof design. While lithium polymer (LiPo) batteries offer excellent energy-to-weight performance, their thermal instability and flammability make them less favorable for internal or ingestible applications [87,88].

**Figure 4:**
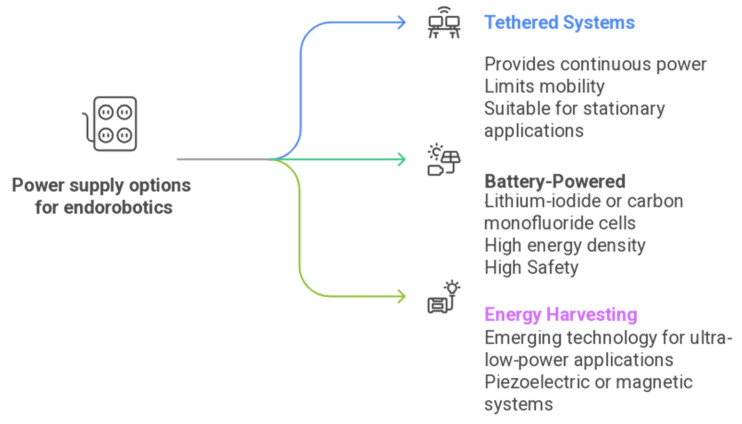
Some power supply options for endorobotics.

Table 7 and Figure 4 show examples of power supply methods for endorobotics that are broadly classified into tethered, battery-powered, and energy-harvesting systems. Tethered designs deliver high and continuous power but compromise mobility, making them suitable for stationary robotic platforms. Battery-based systems, especially those using lithium iodide or carbon monofluoride, offer compact, leak-proof energy sources with high energy density and safety, ideal for ingestible robots. Rechargeable lithium polymer batteries provide superior energy-to-weight ratios but pose flammability risks. To overcome size and longevity limits, novel energy harvesting techniques—such as RF-based backscatter systems, piezoelectric motion harvesters, and MEMS electrostatic devices—are being explored to supplement or replace traditional power solutions [89,90,91].

**Embedded-system optimization and hardware acceleration.**

Emerging approaches to address real-time and energy constraints in AI-enabled endoscopic and endorobotic systems span both algorithmic and hardware levels. At the algorithmic level, techniques such as model pruning, knowledge distillation, and low-bit quantization have been widely explored to reduce memory footprint and power consumption while maintaining near-baseline performance, particularly when combined with hardware-aware training or architecture search strategies tailored to specific edge platforms [95,96,97,98]. In parallel, lightweight convolutional architectures and other compact model designs explicitly co-optimize accuracy, latency, and on-device memory usage, making them more suitable for deployment within endoscopic processors or capsule-based systems.

On the hardware side, increasing attention has been directed toward domain-specific accelerators including embedded neural processing units (NPUs), FPGA-based inference engines, and low-power ASIC implementations to enable real-time inference under stringent size, thermal, and energy constraints. Early studies in gastrointestinal capsule endoscopy and endoscopic imaging workflows suggest that edge-AI acceleration can support clinically acceptable frame rates and latencies either onboard the device or at the endoscopy tower, while reducing dependence on cloud-based processing and improving data privacy [99,100,101]. Collectively, these algorithmic and hardware-level optimizations are emerging as enabling components for safe and practical real-time integration of AI within next-generation endoscopic and robotic screening platforms [102].

### 3.5. Material Considerations

A persistent challenge in endorobotic design is the integration of soft, compliant structures with rigid sensors, actuators, and imaging modules, which introduces trade-offs between flexibility, signal stability, durability, and sterilization compatibility.

#### 3.5.1. Silicone Elastomers

Silicone elastomers are widely used in medical applications due to their exceptional biocompatibility, flexibility, and thermal stability. They can withstand a broad range of temperatures, making them suitable for various sterilization methods, including steam, ethylene oxide, and gamma radiation. However, their hydrophobic nature can lead to protein adsorption and bacterial adhesion, potentially resulting in biofilm formation [103]. To address this, surface modifications such as hydrogel coatings have been developed to enhance hydrophilicity and reduce biofouling.

#### 3.5.2. Thermoplastic Polyurethanes (TPUs)

TPUs offer a balance between flexibility and mechanical strength, making them suitable for components requiring durability and elasticity. They exhibit good resistance to abrasion and can be processed using various manufacturing techniques, including 3D printing. TPUs also demonstrate moderate biocompatibility and can be formulated to meet specific medical requirements [104].

However, material selection should not occur in isolation. For example, hydrogel coatings reduce friction and thus impact both locomotion efficiency and adhesion stability. Likewise, soft elastomers must balance between flexibility and sufficient mechanical support for internal actuation or image sensor stabilization.

## 4. Regulatory Alignment and Translational Validation

### 4.1. System-Level Integration and Trade-Offs

A key engineering challenge is the co-optimization of all subsystems. Locomotion impacts adhesion needs; adhesion affects image stability; imaging and AI drive power demands; and all of the above constrain material and battery choices. This forms a design matrix of interdependencies. For successful translation, endorobotic design must shift from isolated component selection to system-level integration, using iterative design tools, simulation modeling, and feedback from in vitro and in vivo testing.

In summary this section establishes a modular yet interdependent framework for engineering robotic platforms tailored to CRC screening. It emphasizes that no component locomotion, imaging, or adhesion should be optimized in isolation. Instead, the next generation of systems must be designed through concurrent engineering principles, informed by clinical constraints and validated through the pipeline outlined in Section 5.

### 4.2. Regulatory Alignment and Translational Validation Framework

The successful clinical adoption of AI-enabled endorobotic systems in colorectal cancer (CRC) screening requires rigorous validation and regulatory alignment. In this review, we anchor translational readiness to two widely accepted regulatory frameworks: the U.S. FDA’s Total Product Life Cycle (TPLC) model and the principles of Good Machine Learning Practice (GMLP). These frameworks, summarized in Table 8, provide structured guidance for design, validation, authorization, and post-market oversight of AI-enabled medical devices [105].

To improve translational clarity, this review organizes and interprets existing evidence using practical indicators of clinical implementability rather than formal Technology Readiness Level (TRL) scoring. Throughout the manuscript, systems are discussed according to:

(i) Evidence level, ranging from in silico and benchtop studies to preclinical models and early human feasibility trials;
(ii) Validation stage, including reported data on mucosal safety, procedural reliability, and reproducibility;
(iii) Technology maturity considerations, such as workflow integration, sterility and reprocessing feasibility, manufacturability, regulatory progress, and compatibility with existing endoscopy infrastructure.

These indicators reflect how translational readiness is currently evaluated in the endoscopy and medical robotics literature, where heterogeneous study designs and incomplete reporting often preclude formal TRL assignment.

Explainable AI (XAI) methods such as saliency maps, class-activation maps, counterfactual examples, and attention visualization are increasingly explored in medical image analysis to make model decision pathways more transparent to clinicians and support trust-building in high-stakes settings [106,107]. By highlighting image regions or examples that drive predictions and enabling error analysis, these tools can help clinicians verify AI outputs, detect biases, and better understand model limitations, which is central to safe clinical integration [108]. From a regulatory perspective, XAI is closely aligned with broader trustworthy-AI and legal/ethical demands for transparency, accountability, and user-appropriate explanations, and is increasingly recognized as an enabler for regulatory compliance in healthcare AI.

**Table 8:** Alignment of FDA Total Product Life Cycle (TPLC) stages with Good Machine Learning Practice (GMLP) principles relevant to AI-enabled endorobotic colorectal cancer screening systems.

TPLC Stage	Regulatory/Validation Activities	Corresponding GMLP Principles
Design & Development	Initial risk analysis; user-centered engineering; defining intended use; needs-driven innovation to address clinical gaps.	Principle 1—Multidisciplinary expertise across lifecycle. Principle 5—Model design is tailored to intended use. Principle 3—Data representativeness planned at this stage.
Preclinical Validation	In silico testing, bench testing, and in vivo animal studies to confirm basic safety, reliability, and functional performance.	Principle 2—Good software engineering & security practices. Principle 4—Use reference datasets for model training and evaluation
Clinical Trials	Human studies assessing safety (e.g., mucosal trauma), usability, workflow integration, diagnostic performance, ADR, false-positive rate, and real-world variability.	Principle 7—Human–AI team performance. Principle 8—Testing under clinically relevant conditions. Principle 3—Representative patient population
Market Authorization	Regulatory submissions: FDA 510(k), De Novo, PMA; EU MDR documentation; benefit–risk assessment; labeling and transparency requirements.	Principle 10—Clear, essential information provided to users. Principle 2—Software documentation & security. Principle 6—Intended use alignment.
Postmarket Surveillance	Continuous real-world monitoring; incident reporting; drift detection; version control; re-training governance; recalls or updates if needed.	Principle 8—Provide clear, informative user information Principle 9—Ongoing monitoring & re-training risk management. Principle 10—Transparency for updates.

Cybersecurity represents a critical translational consideration for AI-enabled and robotic gastrointestinal endoscopy systems, particularly given their reliance on network connectivity, software updates, and real-time data processing. Current best practice aligns these systems with established medical-device cybersecurity frameworks rather than device-specific endoscopy standards. In the United States, FDA pre-market and post-market cybersecurity guidance for networked medical devices emphasize secure-by-design development, vulnerability management, and coordinated disclosure across the device life cycle [109,110,111]. In the European context, cybersecurity is increasingly treated as a patient-safety requirement through the Medical Device Regulation (MDR), supported by Medical Device Coordination Group guidance and broader digital-security frameworks applicable to connected medical technologies [112,113].

At an international level, standards such as IEC 81001-5-1 define secure development life-cycle processes for health software [114], while the IEC 80001 series [115] addresses security risk management for medical devices operating within hospital IT infrastructures [109,116]. For robotic endoscopy and capsule-robot platforms, these horizontal standards are complemented by established cybersecurity principles from robotic surgery and connected medical systems, including encryption, access control, secure firmware updates, and incident-response planning. Collectively, these requirements underscore that cybersecurity is not an optional add-on, but a core component of clinical readiness and regulatory acceptability for AI-enabled endorobotic screening technologies [110,113].

## 5. Conclusions and Future Priorities

Advancements in endorobotic platforms and artificial intelligence (AI) are poised to redefine colorectal cancer (CRC) screening by offering minimally invasive, highly accurate, and patient-centric alternatives to conventional colonoscopy. This review has highlighted the significant progress made in AI-driven computer-aided detection (CADe) and diagnosis (CADx) systems, as well as in the development of soft robotics, novel locomotion strategies, and enhanced imaging modalities. These technologies collectively aim to increase adenoma detection rates, improve diagnostic precision, and reduce the burden of operator variability and patient discomfort.

Despite this progress, several translational barriers remain. Technical challenges such as limited maneuverability, power constraints, and mucosal safety must be addressed through continued engineering innovation. Regulatory approval and clinical integration are further complicated by the lack of standardized validation frameworks and high-quality, diverse datasets. Moreover, economic considerations and workflow interoperability with current endoscopy practices warrant thorough evaluation.

Real-time implementation of AI systems during colonoscopy also presents several practical constraints that are not consistently reported in the current literature. Most CADe and CADx studies do not provide inference times, frame-by-frame latency, or sustained throughput metrics, making it difficult to determine whether current models can reliably operate at the 25–60 frames-per-second rates required for routine endoscopic video. Furthermore, many high-performing algorithms depend on workstation-grade GPUs or cloud-based processing, which cannot be directly embedded into endoscopic hardware due to physical, thermal, and power limitations. These factors collectively underscore the need for standardized reporting of runtime performance, hardware dependencies, and energy consumption. Future work on model compression, edge-optimized architectures, and embedded AI accelerators will be essential to support true real-time deployment in clinical environments. Emerging approaches such as federated learning, synthetic data generation, and self-supervised pretraining are being explored to mitigate data scarcity and improve generalizability, although their application in endoscopic AI remains largely experimental.

Future research should prioritize the development of biocompatible, energy-efficient robotic systems capable of safe autonomous navigation, coupled with explainable AI models, validated in diverse populations. Key priorities for future research include multicenter prospective validation, cost-effectiveness assessment, integration into population-based screening programs, and regulatory-ready evidence generation. Large-scale clinical trials, interdisciplinary collaboration, and robust regulatory engagement will be critical in translating these innovations from experimental prototypes to routine clinical tools.

## Figures and Tables

**Table 3:** Categories of Locomotion Mechanisms of endorobotics.

Locomotion Type	Principle	Advantages	Limitations	Translational Implications
Peristaltic (biomimetic)	Segmental contraction inspired by earthworm locomotion.	Smooth, tissue-conforming motion; low trauma.	Complex actuation; slow speed; miniaturization challenge.	Promising for atraumatic navigation, but power and complexity limit scalability.
Ambulatory (legged)	Walking using mechanical legs or arms.	High adaptability to terrain; precise positioning.	Mechanically complex; risk of mucosal trauma.	Useful for precision tasks but clinically limited by safety concerns.
Rolling/Wheeling	Continuous rotation of wheels or treads.	Fast locomotion; stable on straight segments.	Poor performance in sharp bends; potential abrasion of mucosa.	Simple and efficient but best suited for short or straight colonic paths.
Magnetic (external actuation)	Movement via external magnetic fields.	Wireless control; avoids onboard propulsion systems.	Control precision limited; performance affected by anatomy variability.	Clinically feasible with external magnet systems; already tested in capsule endoscopy.
Immobile (passive)	Propelled externally (manual push or magnetic drag).	Low onboard complexity; passive imaging/diagnostics possible.	No active control; limited maneuverability in complex anatomy.	Suitable for capsule diagnostics but not for therapeutic interventions.

**Table 5:** Advanced Imaging Technologies in Endoscopy and Their Translational Relevance.

Technology	Clinical Utility	Translational Implications	Reference
CMOS sensors	Enable detection of biological markers (e.g., proteins, cells) relevant to polyp characterization and differentiation.	Foundation for compact, high-resolution endorobotic platforms; allows integration of AI-based tissue classification.	[77]
Narrow Band Imaging (NBI)	Enhances specificity in identifying dysplastic lesions and adenomas.	Already incorporated into commercial endoscopy; provides benchmark for validating AI-based optical biopsy.	[78]
Confocal Laser Endomicroscopy (CLE)	Provides “optical biopsy” with high sensitivity for lesions <5 mm; distinguishes neoplastic from non-neoplastic tissue.	High diagnostic accuracy but limited adoption due to cost and complexity; potential role in targeted robotic platforms.	[79]
Optical Coherence Tomography (OCT)	Detects submucosal invasion; enables monitoring of healing and tissue microstructure.	Adds depth resolution; integration into endorobotics could support minimally invasive staging.	[80]
Hyperspectral/Multispectral Imaging	Identifies molecular signatures; differentiates hyperplastic from dysplastic lesions.	Promising research tool for AI-driven molecular endoscopy; limited by data processing and hardware miniaturization.	[81]

**Table 6:** Estimated Power Demand of Key Components in Wireless Endorobots *.

Component	Estimated Power Consumption	Reference
DC micromotors (inchworm locomotion)	Up to 2 W	[82]
HD CMOS Image Sensor	~0.5 W	[83]
ARM Cortex Microcontroller	~0.3 W	[84]
Wireless Transmission (Wi-Fi/Bluetooth)	~0.5 W	[85]
Real-time Video Streaming	1–2 W	[86]

* Power values represent approximate ranges reported across endoscopic, capsule, and medical robotic systems in the literature; exact consumption varies by design, duty cycle, and operating mode.

**Table 7:** Battery Technologies for endorobotics.

Battery Type	Key Characteristics	
Lithium Iodide (Li/I_2_)	Common in medical implants due to long life, low self-discharge, and chemical stability. Used in pacemakers and neurostimulators.	[92]
Lithium Carbon Monofluoride (Li/CFx)	High energy density (~560–720 Wh/kg), long shelf-life, non-leaking, suitable for capsule endoscopy; downside: low rate capability and heat during discharge	[93]
Lithium Polymer (LiPo)	High energy-to-weight ratio (~300 Wh/kg), rechargeable, but has safety concerns (thermal runaway, swelling); less favorable for in vivo use	[94]

## Data Availability

No new data were created or analyzed in this study.
